# Stathmin Regulates Keratinocyte Proliferation and Migration during Cutaneous Regeneration

**DOI:** 10.1371/journal.pone.0075075

**Published:** 2013-09-16

**Authors:** Sabrina Schmitt, Kai Safferling, Kathi Westphal, Manuel Hrabowski, Ute Müller, Peter Angel, Lars Wiechert, Volker Ehemann, Benedikt Müller, Stefan Holland-Cunz, Damian Stichel, Nathalie Harder, Karl Rohr, Günter Germann, Franziska Matthäus, Peter Schirmacher, Niels Grabe, Kai Breuhahn

**Affiliations:** 1 Institute of Pathology, University Hospital Heidelberg, Heidelberg, Germany; 2 Institute of Medical Biometry and Informatics, Section Medical Informatics, University Hospital Heidelberg, Heidelberg, Germany; 3 BG-Trauma Center, Ludwigshafen, Department of Hand and Plastic Surgery, University of Heidelberg, Heidelberg, Germany; 4 Deutsches Krebsforschungszentrum, Division of Signal Transduction and Growth Control, Heidelberg, Germany; 5 Division of Pediatric Surgery, University Hospital Heidelberg, Heidelberg, Germany; 6 Center for Modeling and Simulation in the Biosciences (BIOMS), University of Heidelberg, Heidelberg, Germany; 7 Biomedical Computer Vision Group (BMCV), BIOQUANT and IPMB, University of Heidelberg and DKFZ, Heidelberg, Germany; Université de Technologie de Compiègne, France

## Abstract

Cutaneous regeneration utilizes paracrine feedback mechanisms to fine-tune the regulation of epidermal keratinocyte proliferation and migration. However, it is unknown how fibroblast-derived hepatocyte growth factor (HGF) affects these mutually exclusive processes in distinct cell populations. We here show that HGF stimulates the expression and phosphorylation of the microtubule-destabilizing factor stathmin in primary human keratinocytes. Quantitative single cell- and cell population-based analyses revealed that basal stathmin levels are important for the migratory ability of keratinocytes *in vitro*; however, its expression is moderately induced in the migration tongue of mouse skin or organotypic multi-layered keratinocyte 3D cultures after full-thickness wounding. In contrast, clearly elevated stathmin expression is detectable in hyperproliferative epidermal areas. *In vitro*, stathmin silencing significantly reduced keratinocyte proliferation. Automated quantitative and time-resolved analyses in organotypic cocultures demonstrated a high correlation between Stathmin/phospho-Stathmin and Ki67 positivity in epidermal regions with proliferative activity. Thus, activation of stathmin may stimulate keratinocyte proliferation, while basal stathmin levels are sufficient for keratinocyte migration during cutaneous regeneration.

## Introduction

Binding of growth factors, cytokines, and chemokines to their respective receptors and subsequent activation of signaling pathways play a pivotal role in the regulation of cellular responses under physiological and pathophysiological conditions. Importantly, most ligands can modify a variety of biological effects in distinct cell types. For example, activation of generic growth factor signaling cascades supports both proliferation and migration of epithelial cells [1]. The relevant mechanisms discriminating between mutually exclusive processes such as mitosis and migration have not been fully defined; however, first data indicate that e.g., ligand concentrations may play an important role in this context [2].

Cutaneous wound healing represents a paradigm for growth factor-induced spatio-temporal regulation of cell mitosis and mobility. After wounding, proliferating epidermal keratinocytes are located in very close proximity to migrating keratinocytes, which is essential for efficient production of new epidermis and fast wound closure [3]. This tightly regulated process partly depends on a double paracrine feedback mechanism initiated by active secretion of keratinocyte-derived interleukin-1 (IL-1), which in turn induces the expression of fibroblast-derived growth factors including keratinocyte growth factor (KGF), granulocyte-macrophage colony-stimulating factor (GM-CSF) and hepatocyte growth factor (HGF; synonym: scatter factor) in an AP-1-dependent manner [4-6]. These factors stimulate keratinocyte mitosis in hyperproliferative areas of the skin, while adjacent epidermal cells in the so-called migration tongue actively cover the injured tissue. The central role of this paracrine feedback communication between fibroblasts and keratinocytes has been documented in genetically modified animals, demonstrating that KGF-, GM-CSF-, or HGF-induced signaling is of pivotal importance for proper reepithelialization during wound closure [7-9].

Binding of HGF to its respective receptor tyrosine kinase c-Met induces proliferation and migration in different epithelial cell types [10]. HGF expression and pathway activation rapidly increase after injury of e.g. liver or lung and may therefore act as a general early response factor upon tissue damage. During cutaneous regeneration, HGF is immediately produced by dermal fibroblasts and stimulates both keratinocyte proliferation and migration *in vitro* and *in vivo* [7,11]. The central role of the HGF/c-Met axis for cutaneous regeneration is supported by the fact that HGF improves wound closure after administration to patients with chronic leg ulcers [12].

At the molecular level, phosphorylation of c-Met upon ligand binding leads to the interaction of signal transducers such as Grb-2 and Shc with receptor multi-substrate docking sites, followed by activation of the Ras/MAPK and PI3K/AKT pathways [10]. In order to define the HGF-dependent regulatory response controlling epithelial cell migration, time-resolved microarray gene expression signatures of primary human keratinocytes were analyzed by inverse modelling after HGF stimulation [13]. These results revealed a dynamic gene regulatory network triggering a responsive state of keratinocytes required for the initiation and maintenance of cell migration. The functional relevance of central HGF network constituents with early and delayed expression signatures involved has been confirmed experimentally for EGFR, uPAR, and CEACAM-1 [5,13]. Interestingly, supplementation of HGF to heterologous co-cultures of primary human keratinocytes and murine fibroblasts lacking c-jun/AP-1 activity partly restored the proliferative response of keratinocytes [14]. While keratinocytes co-cultured with c-jun-deficient fibroblasts formed small colonies with low numbers of vital cells, administration of HGF significantly induced cell division and formation of large colonies with high numbers of vital keratinocytes. Together, these results suggested that the HGF/c-Met signaling axis may provide responsive states necessary for efficient proliferation or migration.

In this study we aim to define how HGF/c-Met signaling affects epithelial cell biology during cutaneous regeneration, a process, which strictly relies on tight spatio-temporal regulation of both cell division and initiation of cell migration. We show that Stathmin (synonym: oncoprotein-18, OP-18) is positively regulated by HGF at transcriptional and posttranslational levels in primary human keratinocytes. Interestingly, *in vitro* and *in vivo* results demonstrate that basal levels of Stathmin support migration, while increased Stathmin concentrations as well as phosphorylation predominantly support keratinocyte proliferation during cutaneous regeneration. Thus, induction of Stathmin may represent a molecular switch triggered by HGF/c-Met signaling, promoting a proliferative phenotype of epidermal cells upon regenerative stimuli.

## Results

### HGF induces Stathmin expression and phosphorylation in human keratinocytes

Microtubule (MT) dynamics are of central relevance for cell proliferation and migration in different cell types and may therefore be of importance for cell division and cell movement [15]. Because the HGF/c-Met signaling axis affects the activity of the MT-destabilizing protein Stathmin in different tumor cell lines [16,17], the effects of HGF on Stathmin expression and phosphorylation were analyzed in primary human keratinocytes.

**Figure 1:**
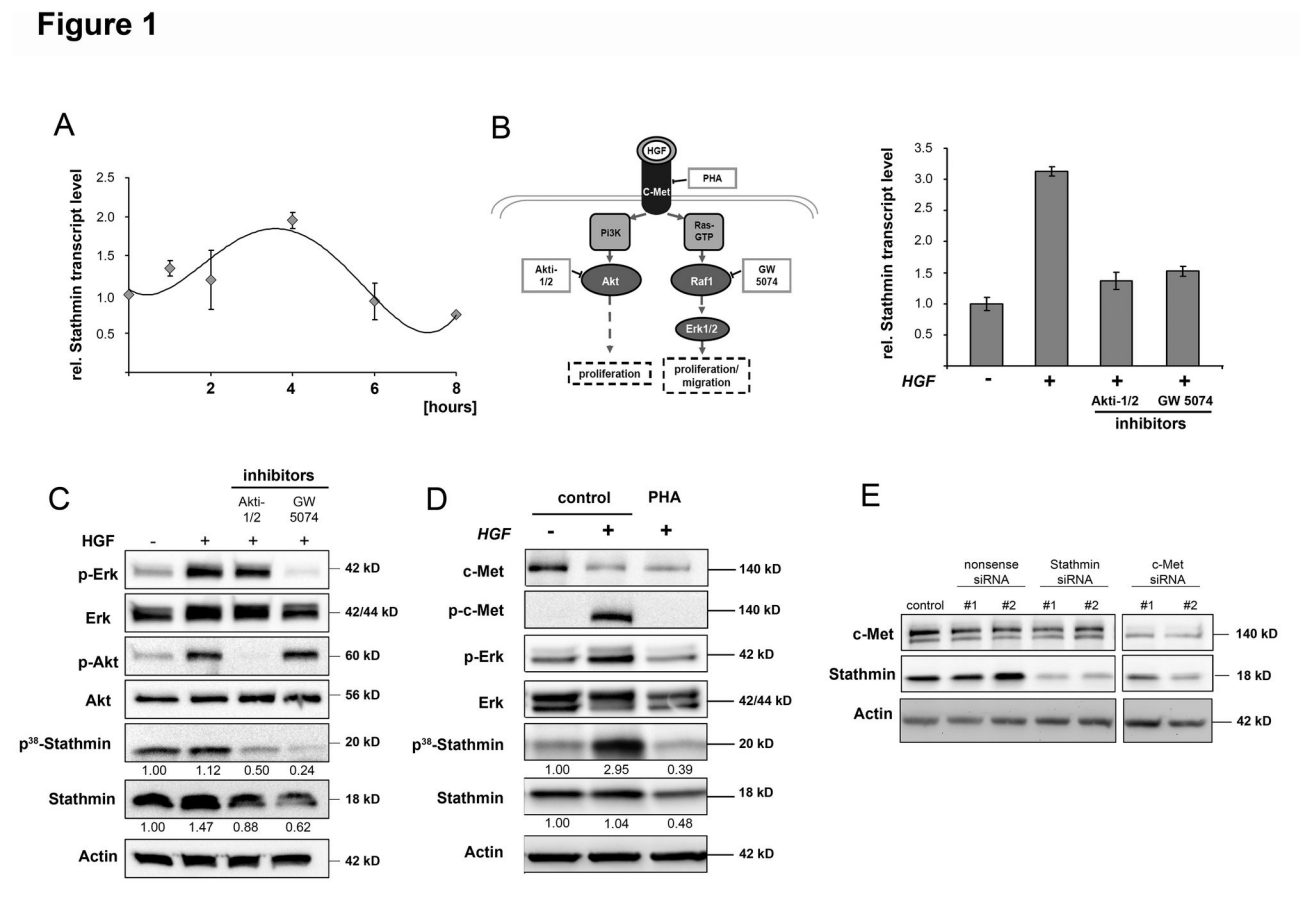
Bimodal activation of Stathmin via HGF/c-Met signaling. (A) Real-time PCR kinetics of Stathmin mRNA expression for 8 hours after administration of HGF (20 ng/ml). For each time-point the ratio of stimulated to untreated primary human keratinocytes was calculated. Data are shown as mean +/- SEM (n=3) and were normalized to transcript levels of untreated cells. (B) Scheme depicting the two major signaling pathways activated by HGF. Chemical inhibitors Akti-1/2 (5 µM) and GW5074 (2 µM) were utilized for selective inhibition of PI3K/AKT- and Ras/Raf/MAPK-pathways. Stathmin transcript levels of keratinocytes were analyzed 1h after stimulation with HGF (20 ng/ml) and administration of both substances for 15 and 30 min, respectively. Data are shown as mean +/- SEM (n=3). (C) Quantitative western immunoblotting analysis of Stathmin and phospho-Stathmin in keratinocytes after stimulation with HGF and administration of Akti-1/2 or GW5074. Total AKT and ERK1/2 as well as phospho-AKT (pAKT) and phospho-ERK1/2 (pERK1/2) served as controls for successful HGF stimulation and pathway inhibition. Numbers indicate quantitative densitometric values of phospho-Stathmin and total Stathmin levels compared to untreated cells. (D) Influence of c-Met inhibition on total Stathmin levels and its phosphorylation status using a receptor-specific inhibitor. Primary keratinocytes were treated with the c-Met inhibitor (PHA-665752, 0.5µM). Numbers indicate quantitative densitometric values of phospho-Stathmin and total Stathmin. (E) Stathmin and c-Met protein expression in keratinocytes after siRNA-mediated inhibition of Stathmin and c-Met after 48 hours (final concentration: 20 nM). Two independent nonsense siRNAs served as negative controls. Different parts of one gel are shown (dividing lines). For all western immunoblots, actin served as loading control.

Quantitative real-time PCR revealed a temporal and moderate increase of Stathmin transcript levels in keratinocytes after HGF administration (Figure 1A). Interestingly, inhibition of the PI3K/AKT and the MAPK-pathways (AKT inhibitor: Akti-1/2; Raf1 inhibitor: GW5074) revealed that both signaling pathways were involved in the HGF-mediated expression of Stathmin (Figure 1B). To confirm the effects of HGF-induced PI3K- and MAPK-pathway activity for Stathmin protein levels, western immunoblotting analysis after administration of Akti-1/2 and GW5074 were performed. While HGF administration induced phosphorylation of AKT and ERK1/2 in primary keratinocytes, inhibitor pre-treatment completely blocked the respective signaling pathways (Figure 1C). In addition, densitometric analysis revealed moderately increased Stathmin protein amounts and phospho-Stathmin levels (at Ser^38^), which were reduced after Akti-1/2 and GW5074 pre-treatment. Signal quantification revealed a more pronounced reduction of phospho-Stathmin than of total Stathmin, indicating that decreasing phospho-Stathmin is not exclusively due to downregulation of total Stathmin (Figure 1C).

In order to test for the HGF/c-Met-dependence of Stathmin after stimulation with HGF in an independent experimental setup, keratinocytes were treated with the c-Met-specific inhibitor PHA-665752 [18]. As already described for HGF/c-Met signaling, total c-Met levels decreased due to ligand-dependent internalization and degradation, acting as a negative feedback loop for pathway attenuation [19] (Figure 1D). Furthermore, total protein and especially phospho-Stathmin levels decreased after PHA-665752 administration (Figure 1D).

Lastly, human keratinocytes were transfected with two independent c-Met-specific siRNAs, leading to an efficient knockdown of c-Met at the protein level (67% and 74%, respectively). Reduction of c-Met expression reduced total Stathmin protein levels in a comparable manner than observed after siRNA-mediated inhibition of Stathmin itself (Figure 1E). HGF administration or perturbation of HGF-induced c-Met signaling did not affect the expression of other MT-destabilizing Stathmin family members such as SCLIP, SCG10, or RB3 (“data not shown”).

Because HGF stimulates the expression of AP-1 constituents such as JunB, Fosl1 (Fra1), and c-Fos [5] and because Stathmin has been demonstrated to be a target gene of AP-1 in embryonic fibroblasts [20], we asked if induction of c-Fos expression may regulate Stathmin expression in keratinocytes. Administration of HGF rapidly induced *c-fos* mRNA expression already after 1 hour, suggesting that c-Fos is a tightly controlled factor essential for early phases of HGF-driven cutaneous regeneration (Figure A in Figure S1). In addition, inhibition of c-Fos expression by siRNAs reduced Stathmin transcript and protein levels compared to nonsense siRNA-transfected cells (Figure B/C in Figure S1).

Together, these results demonstrate that HGF/c-Met signaling not only stimulates Stathmin expression but also affects its phosphorylation in primary human keratinocytes.

### Basal Stathmin levels are important for keratinocyte migration

Because HGF/c-Met signaling triggers a migratory phenotype [7,13] we first analyzed the basal effects of HGF on random keratinocyte motility in short-term experiments (Figure S2). While full media conditions led to the formation of stable and rotating cell colonies, HGF alone induces a migratory phenotype of individual keratinocytes. Full starvation conditions significantly reduced cell migration and (at later time points) induced differentiation and cell death.

**Figure 2:**
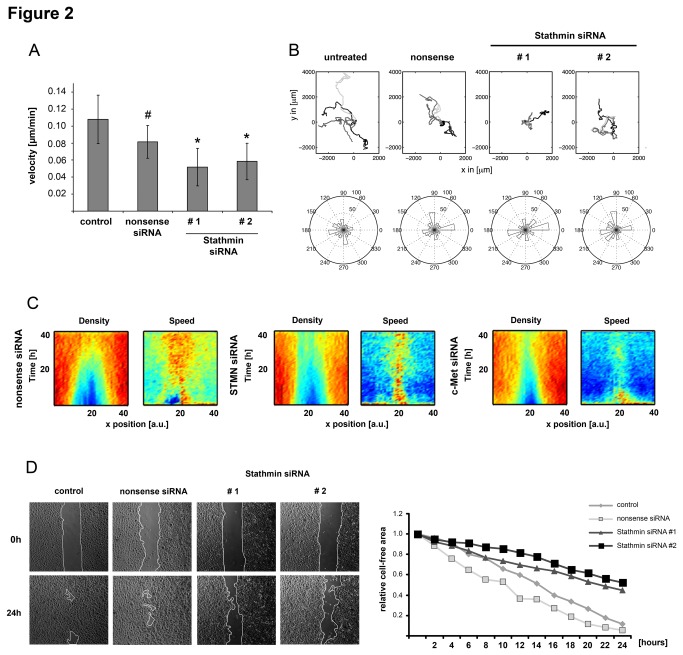
Basal Stathmin supports keratinocyte migration. (A) Manual tracking of individual primary keratinocyte velocity by time-lapse microscopy. Values represent means +/- SEM (n>10). Statistical test: Mann-Whitney U, p*<0.05. # - Nonsense siRNA-transfected cells were used for statistical comparison. (B) Cell trajectories of keratinocytes after centering all starting points to 0,0 (upper panel). Angle statistics depicting distribution of angles during keratinocyte runs (Rose plot). Diameter shows time, while perimeter indicates the angle of cell migration (lower panel). (C) Kinographs showing the cell density (left) and speed profiles (right) during the gap filling process of migrating keratinocytes. Profiles are color-coded and depict all frames (y-axis represents time, x-axis represents cell position). Cell densities are computed in relation to the position across the gap. Color-coding (density): red - high density, blue - low density. Color-coding (speed): red - high speed, blue - low speed. (D) 2D-migration assay of confluent keratinocytes in a 500 µm gap. Migratory ability was documented using time-lapse microscopy. Relative gap closure was exemplarily calculated and relative cell-free areas are depicted for 24 time-points (1: open gap; 0: closed gap). Representative pictures for each biological sample after 24 hours are shown. Migration front is indicated (white line). All experiments have been performed at least two times showing similar results.

Since Stathmin has been described to affect mobility of different cell types *in vitro* [21,22], we aimed to analyze the effect of siRNA-mediated inhibition of basal Stathmin on microtubule dynamics and keratinocyte migration. All functional assays were performed under full media conditions because long-term starvation or even reduction of supplements drastically affected keratinocyte morphology and behavior. Transient transfection of two independent Stathmin-specific siRNAs reduced its expression at the mRNA and protein levels (Figure A/B in Figure S3). As expected, inhibition of a microtubule-destabilizing factor led to increased amounts of polymerized tubulin in keratinocytes (Figure C in Figure S3). Single cell time-lapse microscopy revealed that the average velocity of individual keratinocytes was significantly impaired up to 40% (Figure 2A). Analysis of cell trajectories demonstrated that the loss of Stathmin reduced the distances of free migration; however, the distributions of directions of motion were not affected (Figure 2B).

In order to confirm the pro-migratory effect of Stathmin, the cell density of keratinocytes was analyzed for 48 hours using a 2D-migration assay. Tracking of all individual cells revealed that the inhibition of Stathmin or c-Met reduced the ability of keratinocytes to close a defined gap (Figure 2C). Moreover, the speed of all cells was significantly reduced. Analyzing the migration front showed that the knockdown of Stathmin reduced the velocity of lateral movement up to 41% (Figure 2D). As expected, inhibition of HGF/c-Met signaling by PHA-665752 or receptor-specific siRNAs equally diminished keratinocyte motility (“data not shown”).

Importantly, silencing of Stathmin did not induce compensatory expression of other Stathmin family members such as SCLIP, SCG10, or RB3, which may affect the migratory behavior (“data not shown”). Together these results demonstrate that basal Stathmin levels are sufficient for proper keratinocyte migration.

### Dynamic Stathmin expression in keratinocytes during wound healing

The expression of Stathmin in the epidermis during cutaneous regeneration has not been described so far. In order to define the expression of Stathmin in keratinocytes, its spatio-temporal distribution in normal murine epidermis and during cutaneous regeneration was analyzed. For this reason, C57/Bl6 mice were wounded by punch biopsy and skin specimens were collected at different time points after wounding.

**Figure 3:**
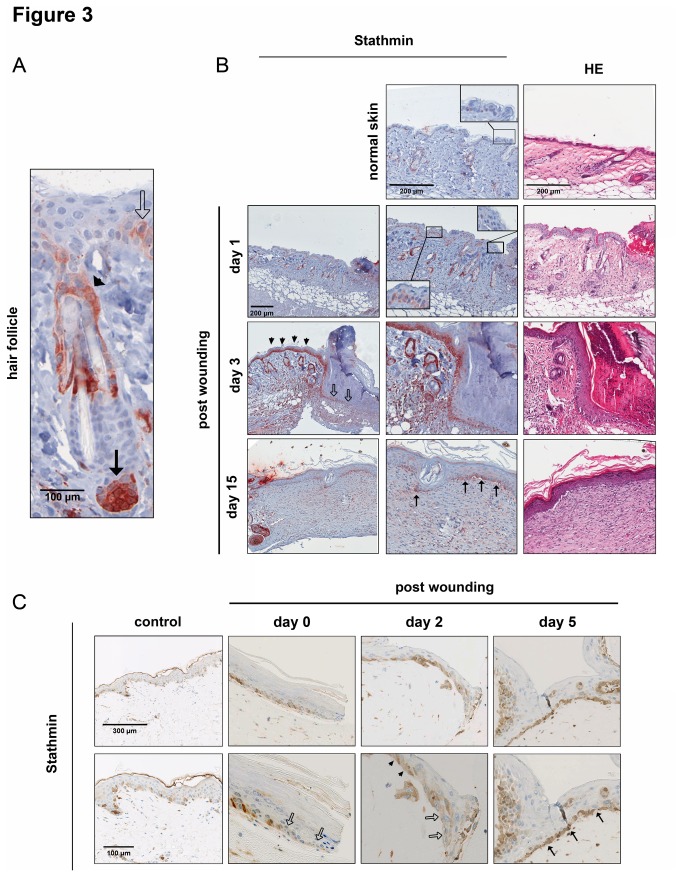
Differential Stathmin expression in skin in wound healing. (A) High-power magnification of hair follicle in unwounded mouse skin after Stathmin staining. Black bordered arrows: Stathmin-positive interfollicular keratinocytes; black arrows: keratinocytes surrounding the papilla; arrowheads: cells encircling the sebaceous gland. (B) Exemplary pictures for Stathmin- and H&E overview staining in unwounded and wounded mouse skin at different time-points after wounding (day 1, 3, and 15 post-wounding). Black bordered arrows: migration tongue; black arrows: keratinocytes after reepithelialization; arrowheads: epidermal keratinocytes with high Stathmin positivity. (C) Stathmin staining of OTC samples consisting of primary human keratinocytes and fibroblasts after including 8 µm punches (2 independent sets of samples were analyzed). Black bordered arrows: migration tongue; black arrows: keratinocytes after reepithelialization; arrowheads: epidermal keratinocytes with high Stathmin positivity.

Immunohistochemistry revealed low-level expression of Stathmin in few basal keratinocytes of the unwounded interfollicular epidermis (Figure 3A). High staining intensity was detected in keratinocytes encircling the sebaceous glands and cells near the papilla of the hair follicle, while no obvious positivity of the outer root sheath keratinocytes was observed. Stathmin expression increased in the basal layers of the epidermis already one day after wounding (Figure 3B). Only a moderate Stathmin induction was detectable in the foremost keratinocytes representing actively migrating keratinocytes (Figure 3B, bordered arrows) [23]. In contrast, highest Stathmin intensity was observed in cells of the adjacent, hyperplastic epidermal regions (Figure 3B, arrowheads). After wound closure, Stathmin levels in basal keratinocytes remained higher in newly formed epidermis as compared to neighboring regions (Figure 3B, black arrows).

In order to exclude the possibility that the lack of Stathmin induction in the epidermal migration tongue was a specific phenomenon of murine wound healing, its expression was analyzed in an organotypic coculture (OTC) model consisting of primary human keratinocytes and fibroblasts after including 8 mm punch wounds [24]. Specimens were collected after different time points and processed for Stathmin staining. Again, low Stathmin abundance was observed in the foremost epidermal regions (Figure 3C, bordered arrows), while elevated protein concentrations were detected in adjacent, mitotically active regions and after wound closure in keratinocytes of the basal layer (exemplarily shown for day 2 and 5 after wounding; Figure 3C, arrowheads and black arrows).

Together, these results demonstrate that during the process of cutaneous regeneration in mice and humans, low-level expression of Stathmin is maintained in the migration tongue, while its expression increases in actively proliferating regions of the epidermis.

### Elevated expression of Stathmin in proliferating keratinocytes

The observed Stathmin staining pattern in murine skin and human OTC samples after wounding as well as the fact that HGF/c-Met signaling has been described to induce keratinocyte proliferation [11], suggested that HGF-stimulated Stathmin may directly support keratinocyte proliferation in this context.

**Figure 4:**
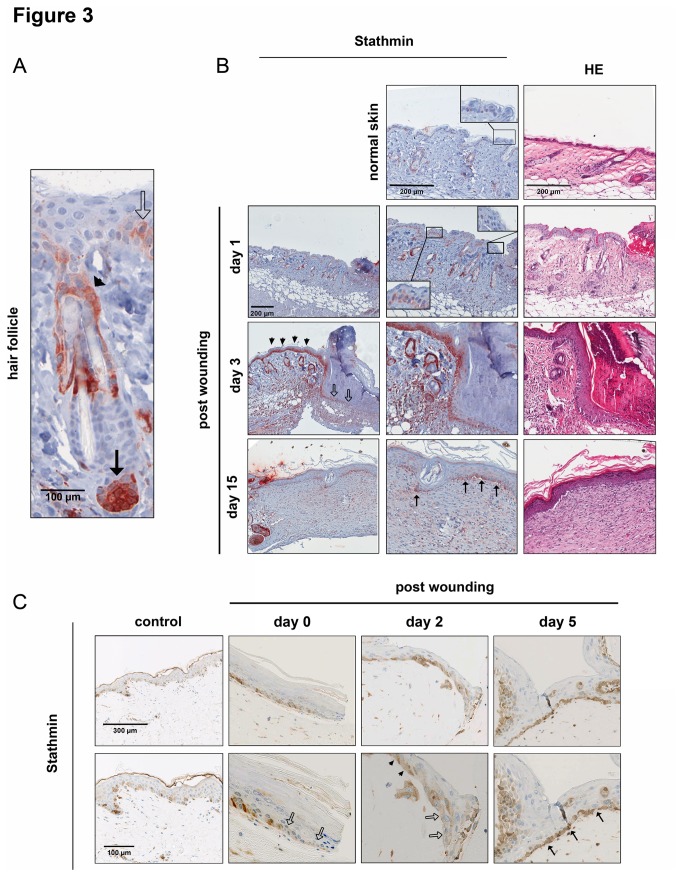
Increased Stathmin levels support keratinocyte proliferation. (A) DNA incorporation assay (SYBR-green assay) after siRNA-mediated inhibition of Stathmin in keratinocytes (final concentration: 20 nM). Nonsense siRNA served as negative controls and was used for statistical comparison (#). Data are shown as mean + SEM (n=3). (B) Keratinocyte viability assay (MTT assay) after siRNA-mediated inhibition of Stathmin (final concentration: 20 nM). Nonsense siRNA served as negative controls and was used for statistical comparison (#). Data are shown as mean + SEM (n=3). (C) Mitotic events of individual keratinocytes were automatically detected after Stathmin or c-Met inhibition based on morphological features. (D) FACS cell cycle analysis. The number of S-phase keratinocytes is indicated. Statistical test: Mann-Whitney U, p*<0.05, p**<0.01, p***<0.001.

To confirm the pro-proliferative role of Stathmin in keratinocytes DNA replication and cell viability were measured after Stathmin silencing. DNA content and viability were reduced in a comparable manner after Stathmin silencing (Figure 4A, B). These data were corroborated by independent FACS analysis and automated, time-resolved and quantitative cell imaging data, demonstrating that the total number of mitotic events was reduced after Stathmin silencing within 48 hours (Figure 4C, D).

Because HGF-induced c-Fos expression affected Stathmin levels in keratinocytes (Figure S1), we asked if c-Fos inhibition may phenocopy the effects observed after siRNA-mediated reduction of Stathmin expression. Accordingly, the knockdown of c-Fos moderately reduced cell migration (-40%) while a more pronounced effect was observed for cell proliferation (-70%), (”data not shown”).

The results collected from primary keratinocytes together with our murine and OTC wound healing data suggest that Stathmin expression predominantly supports keratinocyte proliferation in the wound healing context.

### Elevated expression and phosphorylation of Stathmin correlates with keratinocyte proliferation during wound closure

In order to confirm the *in vivo* relevance of Stathmin for keratinocyte proliferation during wound healing, we systematically correlated its expression with the mitotic activity of keratinocytes in OTC models after wounding. For this, an automated and quantitative approach correlating Ki67 positivity (as measure for keratinocyte proliferation) and Stathmin staining was used [24].

**Figure 5:**
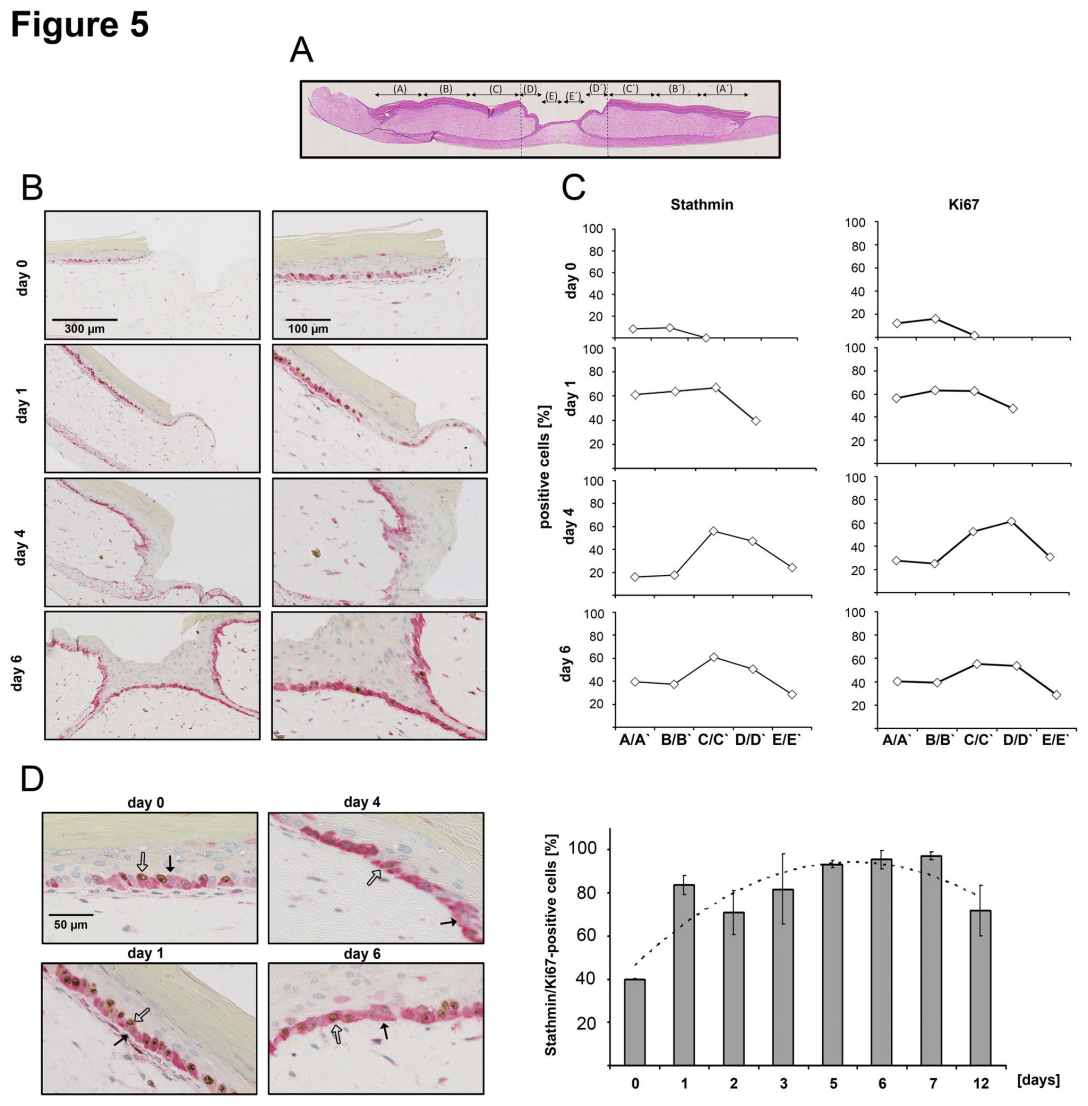
Increased Stathmin levels correlate with keratinocyte proliferating in an OTC wound healing model. (A) Schematic display of sample segmentation for automated analysis of OTC wound specimens. Segmentation in 10 areas was defined using HE-staining, while quantitative evaluation was performed using consecutive sections after Ki67/Stathmin double staining. Opposite areas were pooled (A/A’, B/B’, C/C’, D/D’, E/E’) to increase the cell number suitable for statistical analysis. (B) Ki67 and Stathmin double staining of OTC wound healing sections. Exemplary wound edges were shown for 0 (immediately after wounding), 1, 4, and 6 days after punching. (C) Percentage of Stathmin (left) and Ki67 (right) positive cells in different regions of the OTC specimens at indicated time-points. Please note that the regions E/E' (for day 1) and D/D’; E/E’ (for day 0) are not detectable due to incomplete wound closure at early time points. (D) Exemplarily, Ki67/Stathmin double staining is shown. Results of automated quantitative analysis of Ki67 and Stathmin positivity in keratinocytes of the basal epidermal layer are depicted. Black arrows: Ki67-negative/Stathmin-positive cells; White arrows: Ki67/Stathmin-positive cells. Dashed line in bar graph indicates the mathematical regression for the whole experimental time-course. Two independent time-courses were analyzed showing similar results.

Ki67/Stathmin double staining was performed in two independent series of OTC wound healing kinetics. For spatial quantification, all tissue specimens were divided into 10 areas (5 per wound side: A/A’, B/B’, C/C’, D/D’, E/E’) and Ki67/Stathmin double positive keratinocytes in the basal cell layer were automatically counted for each region (Figure 5A). Quantitative analysis revealed that immediately after wounding the number of Stathmin and Ki67 positive keratinocytes was low in all areas of the epidermis (time-point: 0; Stathmin: up to 9.7%, Ki67: up to 16.1%; Figure 5B, 5C). Already after 1 day, the number of Stathmin and Ki67 positive cells rapidly increased (Stathmin: up to 95.9%, Ki67: up to 62.9%) and remained high until wound closure. After day 4, intensive Stathmin and Ki67 staining was observed in epidermal regions in close proximity to the migration tongue (Stathmin: 50.7%, Ki67: 53.5%), while lower amounts were detected in migratory active cells (Stathmin: 28.7%, Ki67: 28.6%). After wound closure (day 5 to 6) the number of Ki67- and Stathmin-positive keratinocytes remained elevated in the areas of reepithelialized epidermis; however, the total number slightly decreased (Stathmin: 22.0%, Ki67: 23.2%).

To further define how many proliferating keratinocytes were Stathmin positive, the number of Ki67/Stathmin double-positive cells was defined at different time points using newly developed segmentation and registration algorithms. Immediately after wounding, about 40% of proliferating cells stained positive for Stathmin, while the number of double positive cells drastically increased up to 83.6% after 1 day (Figure 5D). The proportion of Ki67/Stathmin positive keratinocytes remained high for at least 7 days (up to 91%) and slightly declined at day 12.

**Figure 6:**
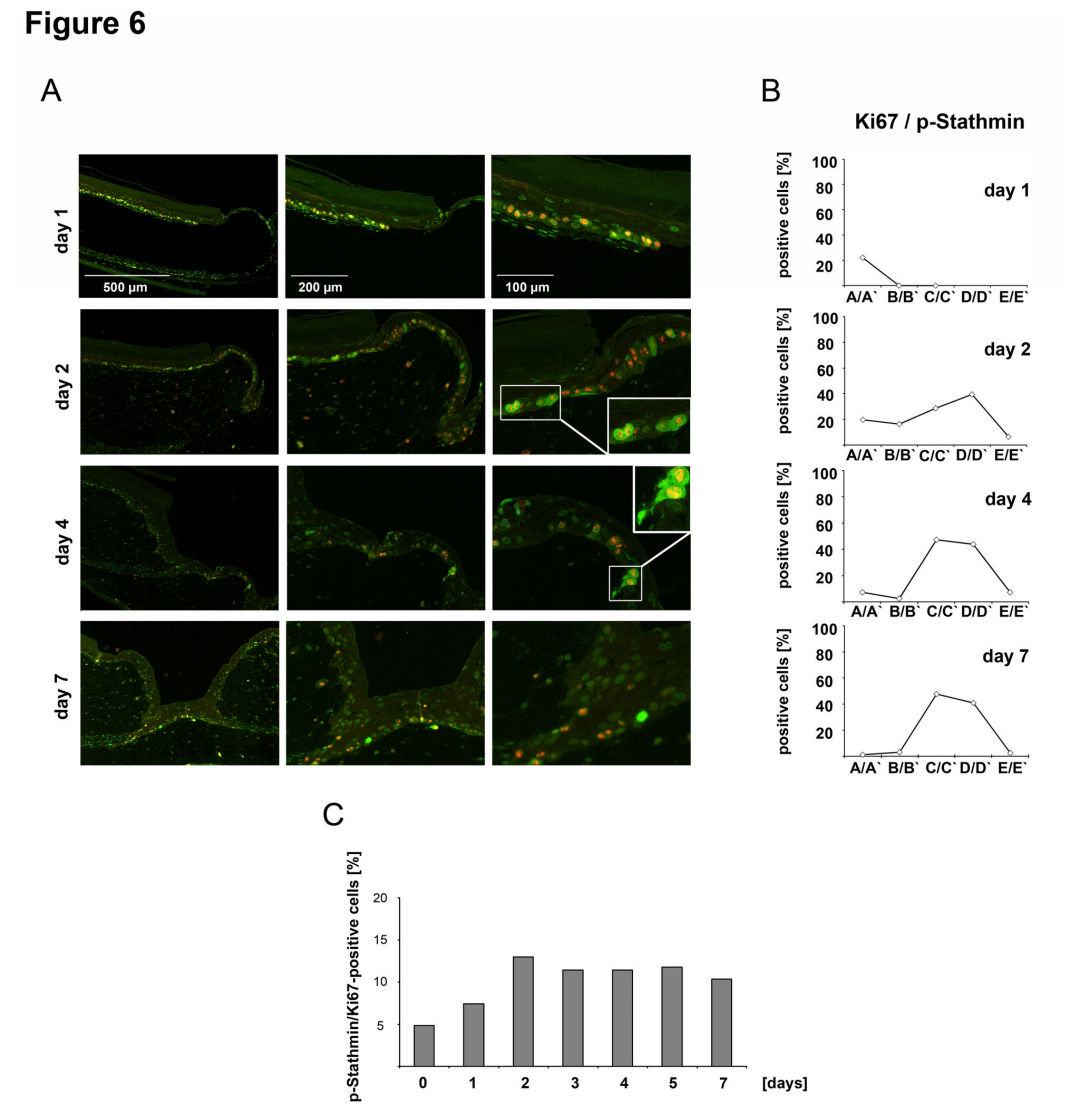
Increased phospho-Stathmin levels correlate with keratinocyte proliferation in an OTC wound healing model. (A) Immunofluorescence Ki67/phospho-Stathmin double staining of OTC wound healing sections. Representative wound edges were shown for 1, 2, 4 and 7 days after punching. For days 2 and 4, Ki67/phospho-Stathmin double positive keratinocytes are highlighted. (B) Percentages of Ki67/phospho-Stathmin positive cells are shown. Note, that regions D/D' and/or E/E' are not detectable due to incomplete wound closure at very early time points. (C) Summarized results of automated quantitative analysis of Ki67 and phospho-Stathmin positivity in keratinocytes of the basal epidermal layer are depicted.

Since initial results demonstrated that HGF not only induced Stathmin expression but also its phosphorylation (Figure 1), we asked if phospho-Stathmin levels were increased and correlated with keratinocyte proliferation in the OTC model. For this reason double immunofluorescence staining for phospho-Stathmin and Ki67 was performed and quantitatively analyzed using the same partitioning approach and mathematical algorithms as described above (Figure 5A). These results revealed that especially at earlier time-points (before wound closure) phospho-Stathmin levels increased in proliferative active areas next to the migratory active regions (Figure 6A, 6B). The number of Ki67/phospho-Stathmin-positive keratinocytes increased; more importantly, double-positive cells were highest in areas in proximity to the migration tongue after 4 days (43.8%). The number of double-positive cells remained high for 7 days after wounding (Figure 6C).

These quantitative data demonstrate that during wound healing increased Stathmin and phospho-Stathmin levels are detectable in mitotically active keratinocytes, while low-level Stathmin abundance and phosphorylation is observed in migratory regions.

## Discussion

Wound healing represents an excellent model for analyzing the impact of specific input signals on mitosis and cell migration. Cutaneous regeneration is a multi-step process, which requires well-orchestrated interaction between epidermal keratinocytes, dermal fibroblasts, as well as infiltrating inflammatory cells and it is known that a network of secreted factors controls communication and functional behavior of keratinocytes leading to efficient wound closure [3,25]. However, proliferation and migration appear to be mutually exclusive states, since the cellular composition (e.g., intermediate filaments) allows the initiation and maintenance of only one process at a given time-point.

This raises the question how keratinocytes may differentially initiate biological responses upon growth factor stimulation. Our data demonstrate that HGF-induced signaling drives keratinocyte proliferation through activation of both Stathmin expression and its phosphorylation. This is supported by the fact that HGF alone is able to efficiently increase keratinocyte proliferation in heterologous coculture systems consisting of murine AP-1-deficient fibroblasts and primary human keratinocytes [5], in human keratinocyte-derived cells [26], or in *in vivo* wound healing models [11].

In addition, the HGF/c-Met signaling axis has been described to induce keratinocyte migration [7]. This is supported by mathematical models for migration and confirmatory experimental data [13,27]. However, inverse modeling of gene networks revealed that initiation and maintenance of HGF-stimulated keratinocyte migration strictly depends on a second input via sequential activation of other tyrosine kinase receptors (e.g., EGFR signaling). Thus, activation of the HGF/c-Met axis triggers a responsive state, which needs subsequent activation of additional signaling pathways typically feeding intracellular MAPK pathway activity to initiate and maintain keratinocyte migration.

Bifunctional properties are not limited to the HGF/c-Met axis but have already been identified for other signaling pathways. In particular, secretion of fibroblast-derived paracrine factors such as GM-CSF, KGF, SDF-1, and HGF is necessary for proper epidermal wound closure by regulating keratinocyte mitosis, migration, and differentiation [3,6,8,28]. For most of these factors positive effects on proliferation as well as migration have been demonstrated *in vitro* and/or *in vivo*. For example, next to the HGF/c-Met pathway, KGF/KGFR signaling is pivotal for both keratinocyte cell division and mobility [7,9,13,29]. First data indicate that adjustment of biological behavior may be based on varying environmental growth factor concentrations as was demonstrated for platelet-derived growth factor (PDGF) signaling [2].

This raises the question which pathway-specific target genes may facilitate the cellular decision discriminating between proliferation and migration. Our results suggest that Stathmin is a target of the HGF/c-Met signaling axis, which predominantly supports keratinocyte proliferation during wound healing. Cytosolic Stathmin belongs to a family of proteins (including SCG-19, SCLIP, RB3) involved in MT dynamics through promotion of so called MT ‘catastrophy’, which describes the transition from MT growth to shrinkage [30]. Additional evidence showed that Stathmin-dependent reorganization of the MT cytoskeleton also affected cell migration [31]. We therefore hypothesize that HGF-induced activation of Stathmin expression is important in the decision-making process of keratinocytes with mutually exclusive biological behavior in spatio-temporal proximity. Our *in vitro*, OTC, and *in vivo* results suggest that basal Stathmin levels are sufficient for cell migration, while its increased expression and phosphorylation predominantly supports keratinocyte proliferation. We therefore suggest that elevated Stathmin expression contributes to HGF-induced proliferative reaction and not cell motility.

Interestingly, activation of the HGF/c-Met pathway not only increased Stathmin protein concentrations but also affected its phosphorylation in keratinocytes. Indeed, Stathmin is phosphorylated in response to several signals including those relevant for mitosis and cell cycle progression [32]. Phosphorylation of Ser^16^, Ser^25^, Ser^38^, and Ser^63^ switch off the MT-destabilizing activity of Stathmin, which is necessary for MT-spindle assembly and entry of mitosis. Therefore, the dynamic phosphorylation and dephosphorylation is pivotal for efficient cell cycle propagation [30]. It is known that Ser^38^-Stathmin phosphorylation is mediated by different kinases such as cycline-dependent kinases (CDKs) or MAPK [30], which can be activated by c-Met [31,33]. Thus, HGF/c-Met signaling affects Stathmin in a bimodal manner: it increases its bioavailability and dynamic activity, both important for efficient initiation and maintenance of proliferation. This is reflected by our OTC analyzes showing elevated expression of Stathmin in the hyperproliferative area as well as increased numbers of pSer^38^-Stathmin positive cells. The total number of pSer^38^-Stathmin-positive keratinocytes was lower than the number of Stathmin-positive ones, since only a fraction of cells was undergoing mitosis at the given time point. It is tempting to speculate that HGF-driven increase of Stathmin levels may represent a prerequisite for its subsequent activation by phosphorylation.

Our results demonstrate a significant impact of HGF on Stathmin abundance and activity; however, it is likely that additional signaling pathways may affect its phosphorylation and expression. Although administration of HGF on keratinocytes led to a moderate but detectable increase of Stathmin expression (Figure 1C), inhibition of the PI3K/AKT or MAPK pathway more drastically reduced Stathmin levels and posttranslational modification, indicating that other growth factor pathways positively influence Stathmin concentration and phosphorylation. This has for example been shown for EGF, which equally stimulates Stathmin phosphorylation [16]. It is therefore likely, that the coordinated activity of different growth factors is essential for an optimized spatio-temporal activity of Stathmin.

## Materials and Methods

### Isolation, culture, and treatment of primary human keratinocytes

Human juvenile foreskin was used for isolation of primary keratinocytes as previously described [34]. Isolated cells were cultured in keratinocyte growth medium (PromoCell, Heidelberg, Germany) supplemented with 0.06 mM calcium, 4 µl/ml bovine pituitary extract, 0.125 ng/ml epidermal growth factor, 5 µg/ml human recombinant insulin, 0.33 µg/ml hydrocortisone, 0.39 µg/ml epinephrine, and 10 µg/ml human transferrin at 37°C in a 5% CO_2_ atmosphere. The study was approved by the Local Ethics Committee of the Medical Faculty Heidelberg (approval number S-1492007). Written consents were signed by patients or legal guardians. This procedure was approved by the Local Ethics Committee.

Keratinocytes grown for less than five passages were seeded 12 or 24 h before treatment to achieve subconfluence (60-70%). Growth factors were purchased from R&D Systems (Minneapolis, MN). For growth factor stimulation, cells were treated with HGF (20-50 ng/ml) and further incubated as indicated. Treatment with the kinase inhibitors was performed immediately before HGF administration for 15 (Akti 1/2, 5 µM, Merck, Darmstadt, Germany) or 30 minutes (GW5074, 2 µM, Sigma-Aldrich, St. Louis, USA), respectively. SiRNA treatment with the HiPerFect transfection reagent was performed according to the manufacturers’ instructions (Qiagen, Hilden, Germany). In brief, 2 x 10^5^ cells were seeded in 6-well plates one day before transfection and HiPerFect/siRNA mixture was administered (final concentration: 20 nM). PHA-665752 was administered 1 h before HGF treatment (0.5 µM, TOCRIS Bioscience, Bristol, UK).

### Sample preparation, real-time PCR, and western immunoblotting

Keratinocytes were harvested at indicated time-points after stimulation and/or siRNA transfection. Total RNA was isolated using the Nucleospin RNA II Kit according to the protocol (Macherey-Nagel, Düren, Germany). For semi-quantitative real-time PCR analyzes, reverse transcription was performed using 1µg total RNA (M-MuLV reverse transcriptase, Fermentas, München, Germany) and PCR reactions with respective primer pairs were analyzed in technical triplicates. Primers used in this study are listed in Table S1. The following cycling program was applied using the ABsolute qPCR mix (ABgene, Cambridge, UK): 95°C for 15 min, followed by 40 cycles of 95°C for 15 s, and 60°C for 60 s (StepOne; Applied Biosystems, Carlsbad, CA, USA). Western immunoblotting analyses and tubulin assays were performed as described [22]. Densitometric quantification of protein amounts was performed via Quantity One software (Bio-Rad, München, Germany). Antibodies and respective dilutions are listed in Table S2.

### Immunohistochemistry and immunofluorescence

For immunohistochemistry, representative 2 µm paraffin sections were mounted on positively charged slides, air-dried in an incubator at 42°C overnight, and deparaffinized in xylene. After rehydration in graded ethanols, microwave pretreatment of slides was performed in 0.01 M sodium citrate (pH 6.0) at 600 W for 3 × 5 min. Afterwards, slides were incubated for another 30 min in 0.01 M sodium citrate (pH 6.0), rinsed two times with distilled water, and then incubated for 4 min in TBS. Afterwards, the anti-Stathmin antibody in TBS was added to the samples for incubation at 4°C overnight. After rinsing with TBS, the bound antibodies were detected using the avidin–biotin complex method (DAKO, Hamburg, Germany) and visualized by DAB staining. Slides were counterstained with haematoxylin. No staining was obtained when PBS was used instead of the primary antibodies. Representative tissue areas were digitally documented using the ScanScope CS Scanner (Aperio, Alton, UK; objectives: 20x PlanApo, NA = 0.75 and 40x PlanApo, NA = 0.90; Olympus, Hamburg, Germany). Picture analysis was performed using the ImageScope^TM^ software (Aperio, Alton, UK)

For immunofluorescence staining, keratinocytes were seeded on coverslips. After 2 days, coverslips were scratched with a sterile pipette-tip and subsequently treated w/o HGF (50 ng/ml). Samples were fixed with ice-cold methanol/acetone, incubated with an anti-Stathmin antibody, and labeled with a secondary Cy3-linked anti-rabbit antibody. After DAPI-counterstaining, representative areas were digitally documented using the Cell^R Imaging Station (Olympus, Hamburg, Germany), containing the IX-81-ZDC microscope and digital camera C10600 ORCA-R^2^ (Hamamatsu, Herrsching am Ammersee, Germany). Samples were visualized using the following objectives: 10x UPLanF, NA = 0.3, 20x UPLanSApo, NA = 0.75, 40x UPLanSApo, NA = 0.95. Analysis was performed using the Cell^R^ software. All antibodies and respective dilutions are listed in Table S2.

### Murine full thickness excisional wounds

Excisional wound healing experiments were performed as previously described [8]. Mice were housed under standard conditions and the procedures for performing animal experiments were in accordance with the principles and guidelines of the ATBW and were approved by the federal state government of Baden-Württemberg (approval numbers: G-81/10, G-129/02). In brief, after shaving the back of 12 weeks old Bl6/C57 female mice, 5 mm in diameter full-thickness excisional wounds were inflicted on the mid-dorsum using biopsy punches (Stiefel, Offenbach, Germany). The wounds were left unsutured and without coverage. Subsequently, animals were housed individually. Three animals were analyzed for each time point (0, 1, 3, 6, 8, 10, 13, and 15 days after wounding). Specimens were fixed in buffered 4% formalin, processed for paraffin embedding, and routinely stained with hematoxylin and eosin (H&E) to define wound closure as well as for Ki67- and Stathmin immunohistology.

### Proliferation and viability assays

For analyzing proliferation after gene-specific inhibition, subconfluent keratinocytes were treated with the respective siRNAs for 24 h. Subsequently, 10^3^ cells were seeded in 96 well plates and incubated for further 24 to 72 h (six technical replicates). Samples were washed with PBS and plates were frozen at -20°C for 1 day. SYBR ^®^Green nucleic acid stain (Molecular Probes, Life Technologies Cooperation, Carlsbad, CA, USA) was diluted 1:7500 in 0.1% Triton X-100/PBS and administered to the thawed cells in the dark for 1 h. After washing with PBS, SYBR ^®^Green incorporation was measured at 497 nm excitation and 520 nm emission in a microplate reader (FLUOStar Omega, BMG Labtech, Ortenburg, Germany). Proliferation was calculated using the standard curve method and normalized against untreated keratinocytes.

For analyzing cell viability via MTT-assay, 2 x 10^3^ cells were seeded in 96 well plates and incubated for further 24 to 72 h (10 technical replicates). Cell viability was analyzed using the 3-(4,5-dimethylthiazol-2-yl)-2,5-diphenyl-tetra-zolium-bromide (MTT, Sigma-Aldrich, USA) assay. After incubation for 2 h, the MTT (0.5 mg/mL in medium) solution was removed and tetrazolium salt was resolved in 100 µL of DMSO/ethanol solution (1:2). Colorimetric measurement was performed at 570 nm using an ELISA reader. The number of viable cells was calculated based on respective standard curves and normalization against controls.

Analyses for cell cycle distribution was performed using a FACS-calibur flow cytometer (Becton & Dickinson, Heidelberg, Germany) equipped with a 488 nm air cooled argon laser. Cell cycle analyses were acquired in Fl-3 in linear mode as previously described [22].

### Migration (single-cell and cell population)

For analysis of single cell migration of keratinocyte cultures with low cell density (5x10^4^), keratinocytes were seeded 24 h before analysis. After growth factor stimulation or siRNA transfection, keratinocytes were analyzed using time-lapse microscopy (Cell^R-Imaging Station with accessory climate chamber at 37°C, 40% humidity, and 5% CO_2_ atmosphere; Olympus). Cells were automatically documented every 20 to 60 min for 24 or 48 h, digitally visualized (objectives: 10x UPLanF/, NA = 0.3, 20x UPLanSApo, NA = 0.75, 40x UPLanSApo, NA = 0.95 (Olympus)), and analysed by the Cell^R^ software (Olympus). Single cell migration was analyzed using the ImageJ software (Wayne Rasband, Research Services Branch, National Institute of Mental Health, Bethesda, Maryland, USA) using the accessory plugin “manual tracking”. Keratinocytes were analyzed for every biological sample and mean distance as well as mean velocities were calculated.

To assess migratory activity of keratinocytes at the cell population level via live cell imaging, 4 x 10^4^ cells were seeded one day prior treatment into culture inserts (500 µm defined, cell-free gap, ibidi, Martinsried, Germany). Cell migration was documented by time-lapse microscopy at 37°C, 5% CO_2_ and 40% humidity using the Cell^R Imaging Station (Olympus, IX-81-ZDC microscope, C10600 ORCA-R^2^camera) every 20 to 60 min for 24 or 48 h. Samples were visualized using the following objectives: 10x UPLanF/, NA = 0.3, 20x UPLanSApo, NA =0.75, 40x UPLanSApo, NA = 0.95 (Olympus) and analysed by the Cell^R^ software (Olympus). Relative migratory activity was defined by calculating the cell-free areas (AxioVisionLE, Carl Zeiss MicroImaging GmbH, Jena, Germany).

### Organotypic coculture (OTC) model

Human EpiDerm full thickness (EFT) *in vitro* skin cultures were obtained from MatTek Corporation (Ashland, MA, USA). Epidermal keratinocytes used for EFT skin cultures were derived from human neonatal foreskin and were equilibrated and cultivated according to the manufacturers’ recommendation. EFT cultivation and specimen processing have been described elsewhere [24]. Samples were wounded using 8 mm circular biopsy punches and further cultured at 37°C in a 5% CO_2_ incubator in EFT maintaining medium (MatTek, Ashland, MA, USA). Wound cultures were cultivated for 10 days and collected at different time points (0, 1, 2, 3, 5, 6, 7, and 12 days) for fixation and paraffin embedding.

Tissue sections were immunohistochemically stained using a fully automated Leica BOND™-Max (Leica Microsystems, Wetzlar, Germany) with the Bond Polymer Refine Detection system (Leica Microsystems, Wetzlar, Germany) according to the manufacturers’ instructions. Two independent consecutive sections of all cultures were stained. Immunohistological sections were scanned and digitized using the virtual microscopy platform Nanozoomer Digital Pathology (NDP; Hamamatsu Photonics). After staining, slides were scanned in three z-layers with a spacing of 2 µm each, using a resolution of 460 nm/pixel (20x).

### Bioinformatics and statistics

For the display of the trajectories, data were rescaled in order to set the initial cell position to the origin for all tracked cells. The average run length, average speed, run length, and speed distributions as well as the distribution of moving angles and time dependence of the mean square displacement were computed.

To further define the single-cell migration of all individual cells of the 2D migration assays, we developed an image analysis method, which comprises cell segmentation, tracking, and quantification. For cell segmentation, a top-hat transform and a region adaptive thresholding approach were used. Subsequently, the segmentation result was refined by splitting up clusters of cell nuclei based on a Euclidean distance transform followed by a watershed transform. For cell tracking, we used an extension of the two-step approach consisting of (1) correspondence finding and (2) mitosis detection. Correspondences were determined based on a cost function which combines three criteria: the Euclidean distance of the cell nuclear centroids, the feature similarity of cell nuclei (e.g., mean intensity, area), and the local smoothness of trajectories. After establishing one-to-one correspondences, mitosis events were identified and the respective trajectories were merged, resulting in cell lineage trees. For mitosis detection, mitosis likelihoods were computed based on morphological features of the potential mother and daughter cells [35]. Finally, obvious trajectory breaks (i.e. tracks ending in one frame and re-appearing a few frames later) were automatically detected and merged. The obtained cell lineage trees allowed quantification of the cell motility (velocity, direction) and cell proliferation (mitotic index).

Cell density and speed profiles were calculated based on the cell positions from automated single cell tracking of the scratch assay. For the density profiles, the number of cells within intervals along the x-axis [x,x+Δx] were summed up. Speed was computed from two consecutive frames. Speed and angle profiles were obtained by averaging the values of all cells in an interval [x,x+Δx]. The ratio between the cell density in the cell-free area, and the average cell density in the adjacent regions were computed. This yields a percentage of gap coverage for each frame. To quantify cell mobility the speed of gap coverage through linear regression to the gap filling dynamics was calculated. Statistical analysis of the single cell tracking data was performed using Matlab (version 7.12, MathWorks).

Persistence is computed based on the trajectories of the single cells as

$persistence=\frac{d({\bar{x}}_{0},{\bar{x}}_{end})}{l_{path}}\cdot \sqrt{t_{path}}$, whereby, $d({\bar{x}}_{0},{\bar{x}}_{end})$denotes the (Euklidean) distance between start and end point of the trajectory, *l*_*path*_ the length of the trajectory, and *t*_*path*_ the number of time points for which coordinates are available for this trajectory. The correction factor involving allows to compare trajectories of different lengths. *t*_*path*_ For quantitative analysis of Stathmin, phospho-Stathmin, and Ki67 staining in the OTC cultures, image processing algorithms were developed and implemented using the Visiomorph software (VisioMorph, Visiopharm, Denmark). For proliferation analysis we performed an image pre-processing step using a median filter and applied a Bayesian classifier for nuclei detection. The following post-processing steps have been performed to identify DAPI-positive nuclei: (i) Particles beyond 3 pixels per particle were removed (ii). Structures showing a circularity value of less than 2 were identified as potential nuclei (iii). Conglomerated nuclei (>60 pixels per area) were separated (iv). Separated conglomerates were revised and tested for size and circularity; objects with circularity > 2.5 or a size smaller than 4 pixels were removed. The number of counted nuclei was normalized by the area of the epithelium. For spatial analysis of the tissue sections, specimens were divided in 10 regions (A/A', B/B', C/C', D/D', E/E'). The Stathmin staining and proliferation were calculated separately for each region in each sample.

Data are presented as mean +/- standard deviation. The Spearman rank coefficient was used as a statistical measure of association. The statistical comparison between two groups (unpaired and paired values) was accomplished with the non-parametric Mann-Whitney U test. The significance levels were defined as *p<0.05, **p<0.01, and ***p<0.001 (SPSS software). n.s.: not significant.

## Supporting Information

Figure S1 **HGF-induced c-fos regulates Stathmin expression.** (A) Real-time PCR kinetic of c-fos mRNA for 8 hours after administration of HGF (20 ng/ml). For each time-point the ratio of stimulated to untreated primary human keratinocytes is shown. Data are shown as mean +/- SEM (n=3) and were normalized to transcript levels of untreated cells. (B) Stathmin transcript levels in keratinocytes after siRNA-mediated inhibition of Stathmin or c-fos (final concentration: 20 nM). Two independent c-fos siRNAs were used to confirm the effect on Stathmin expression. (C) Western immunoblotting analysis of total Stathmin levels after inhibition of Stathmin (control) or c-fos by siRNA. For real-time PCR analyzes 18S-RNA was used for calibration, while actin served as loading control for the western blot.
(TIF)

Figure S2 **Effects of HGF on keratinocyte motility.** Long-term starvation of keratinocytes (even with HGF) induces differentiation and apoptosis. In order to test for the immediate effects of HGF (within 24 hours), an experimental setup was used where cells are cultured under full media (control) or under starvation (+/- HGF) conditions. Migration of 20-30 cells was monitored for 24 hours and analyzed using bioinformatics with regard to (A) trajectories, (B) speed and persistence, (C) mean square displacement (see Materials and Methods).
Cells in complete medium are motile and show intact adherence to neighboring cells. Loss of attachment and migration of individual cells is rare. This can be quantified by a relatively low average speed and low directional persistence. The mean square displacement grows with time for a range up to 5 minutes, after which it is nearly constant with increasing time. This reflects confined motion as cells do not detach from cell clusters. Cells, which were starved and treated with HGF show loss of adherence and strong motility, characterized by high speed and directional persistence. Here, the mean square displacement grows linearly with time. Starved cells migrate individually but very slowly. Directional persistence is comparable to HGF-treated cells, mean square displacement grows linearly, but very slowly, and speed is reduced, even in contrast to cells grown in complete medium. For all treatments, speed per frame is exponentially distributed, and moving angles (direction of motion) show uniform distribution (no preference; data not shown).
(TIF)

Figure S3 **Efficient knockdown of Stathmin transcripts and protein levels.** (A) Stathmin transcript levels in primary human keratinocytes were measured by semi-quantitative real-time PCR and (B) western immunoblotting after transient transfection of two Stathmin-specific siRNAs (#1, #2; 20 nM each) for 24 h. Nonsense-transfected cells were used as controls. For normalization, 18S-rRNA and actin were used, respectively. Values represent means +/-SEM; (n=3). (C) The tubulin assay revealed an accumulation of polymerized ß-tubulin for both Stathmin-specific siRNAs as compared with nonsense siRNA-transfected cells. Treatment with microtubule stabilizing paclitaxel (1µM) served as control for tubulin polymerization, while vinblastine (1 µM) served as control for depolymerization. Ratios between soluble (sol.) and polymerized (pol.) tubulin are indicated (high values: low degree of polymerization; low values: high degree of polymerization).
(TIF)

Table S1 **Sequences of primers and siRNAs used in this study.**
(DOCX)

Table S2 **List of antibodies used in this study.**
(DOCX)
