# Correction: Stathmin Regulates Keratinocyte Proliferation and Migration during Cutaneous Regeneration

**DOI:** 10.1371/annotation/1ece268f-578b-429a-b70b-c3ff002aec0b

**Published:** 2013-12-13

**Authors:** Sabrina Schmitt, Kai Safferling, Kathi Westphal, Manuel Hrabowski, Ute Müller, Peter Angel, Lars Wiechert, Volker Ehemann, Benedikt Müller, Stefan Holland-Cunz, Damian Stichel, Nathalie Harder, Karl Rohr, Günter Germann, Franziska Matthäus, Peter Schirmacher, Niels Grabe, Kai Breuhahn

Figure 4 is incorrect. Please view the correct figure 4 here: http://plosone.org/corrections/pone.0075075.g004.cn.tif

