# Supplementary figures and images for: Stathmin Regulates Keratinocyte Proliferation and Migration during Cutaneous Regeneration
Source: PLoS One. 2013 Sep 16;8(9):e75075. doi: 10.1371/journal.pone.0075075 (PMC3774809; doi:10.1371/journal.pone.0075075)

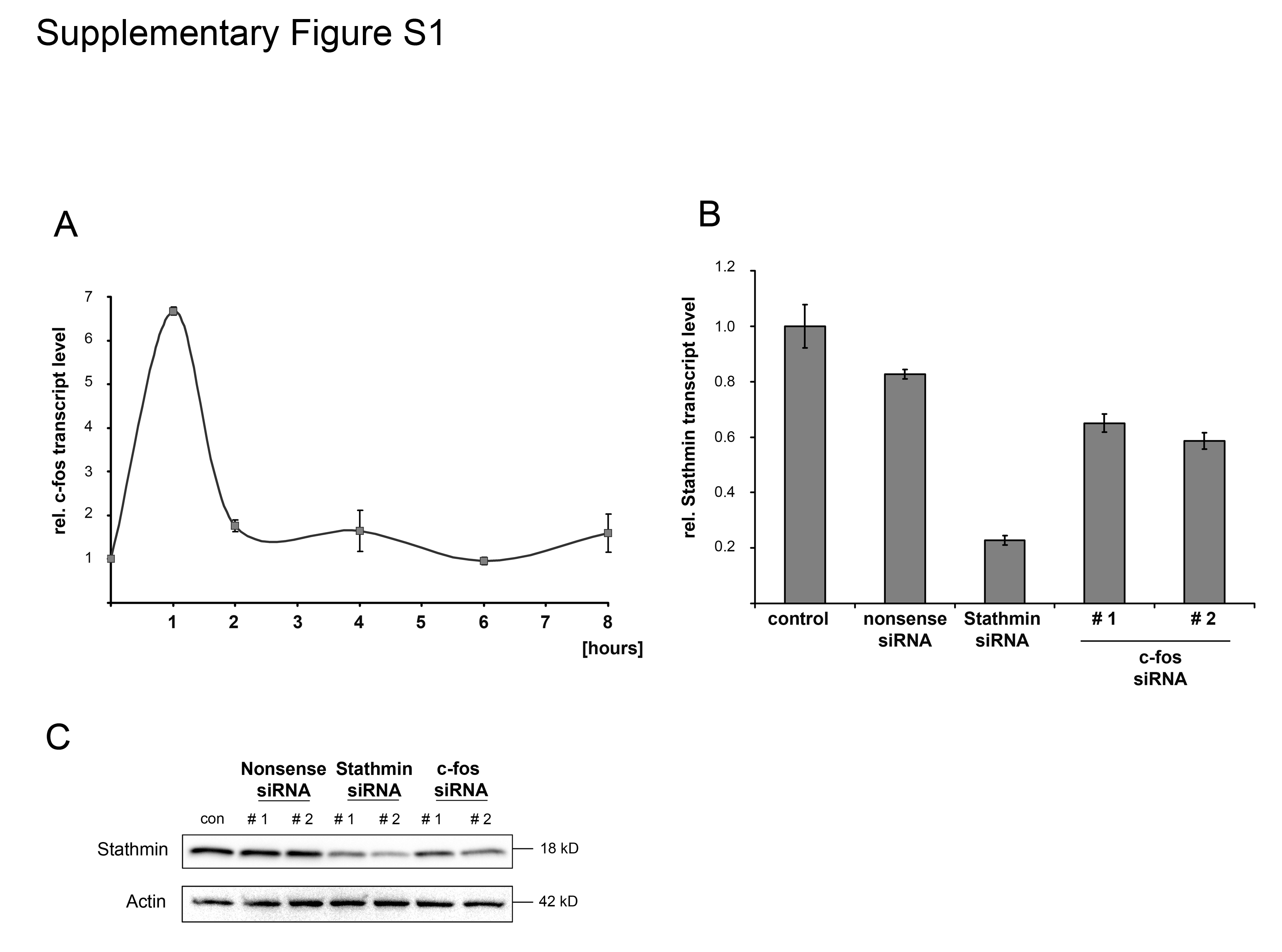

Supplement: Figure S1 — HGF-induced c-fos regulates Stathmin expression. (A) Real-time PCR kinetic of c-fos mRNA for 8 hours after administration of HGF (20 ng/ml). For each time-point the ratio of stimulated to untreated primary human keratinocytes is shown. Data are shown as mean +/- SEM (n=3) and were normalized to transcript levels of untreated cells. (B) Stathmin transcript levels in keratinocytes after siRNA-mediated inhibition of Stathmin or c-fos (final concentration: 20 nM). Two independent c-fos siRNAs were used to confirm the effect on Stathmin expression. (C) Western immunoblotting analysis of total Stathmin levels after inhibition of Stathmin (control) or c-fos by siRNA. For real-time PCR analyzes 18S-RNA was used for calibration, while actin served as loading control for the western blot. (TIF) [file pone.0075075.s001.tif]

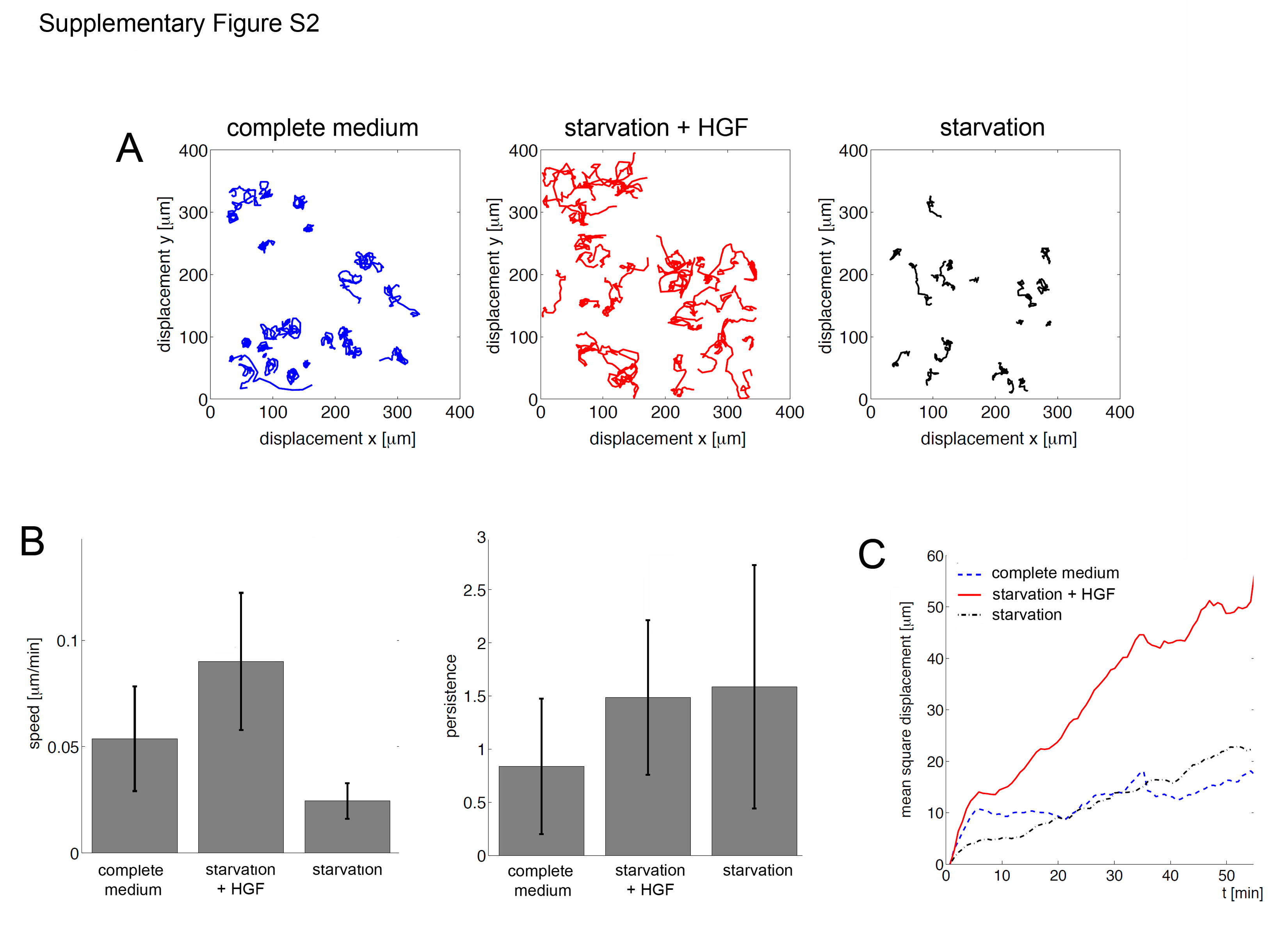

Supplement: Figure S2 — Effects of HGF on keratinocyte motility. Long-term starvation of keratinocytes (even with HGF) induces differentiation and apoptosis. In order to test for the immediate effects of HGF (within 24 hours), an experimental setup was used where cells are cultured under full media (control) or under starvation (+/- HGF) conditions. Migration of 20-30 cells was monitored for 24 hours and analyzed using bioinformatics with regard to (A) trajectories, (B) speed and persistence, (C) mean square displacement (see Materials and Methods). Cells in complete medium are motile and show intact adherence to neighboring cells. Loss of attachment and migration of individual cells is rare. This can be quantified by a relatively low average speed and low directional persistence. The mean square displacement grows with time for a range up to 5 minutes, after which it is nearly constant with increasing time. This reflects confined motion as cells do not detach from cell clusters. Cells, which were starved and treated with HGF show loss of adherence and strong motility, characterized by high speed and directional persistence. Here, the mean square displacement grows linearly with time. Starved cells migrate individually but very slowly. Directional persistence is comparable to HGF-treated cells, mean square displacement grows linearly, but very slowly, and speed is reduced, even in contrast to cells grown in complete medium. For all treatments, speed per frame is exponentially distributed, and moving angles (direction of motion) show uniform distribution (no preference; data not shown). (TIF) [file pone.0075075.s002.tif]

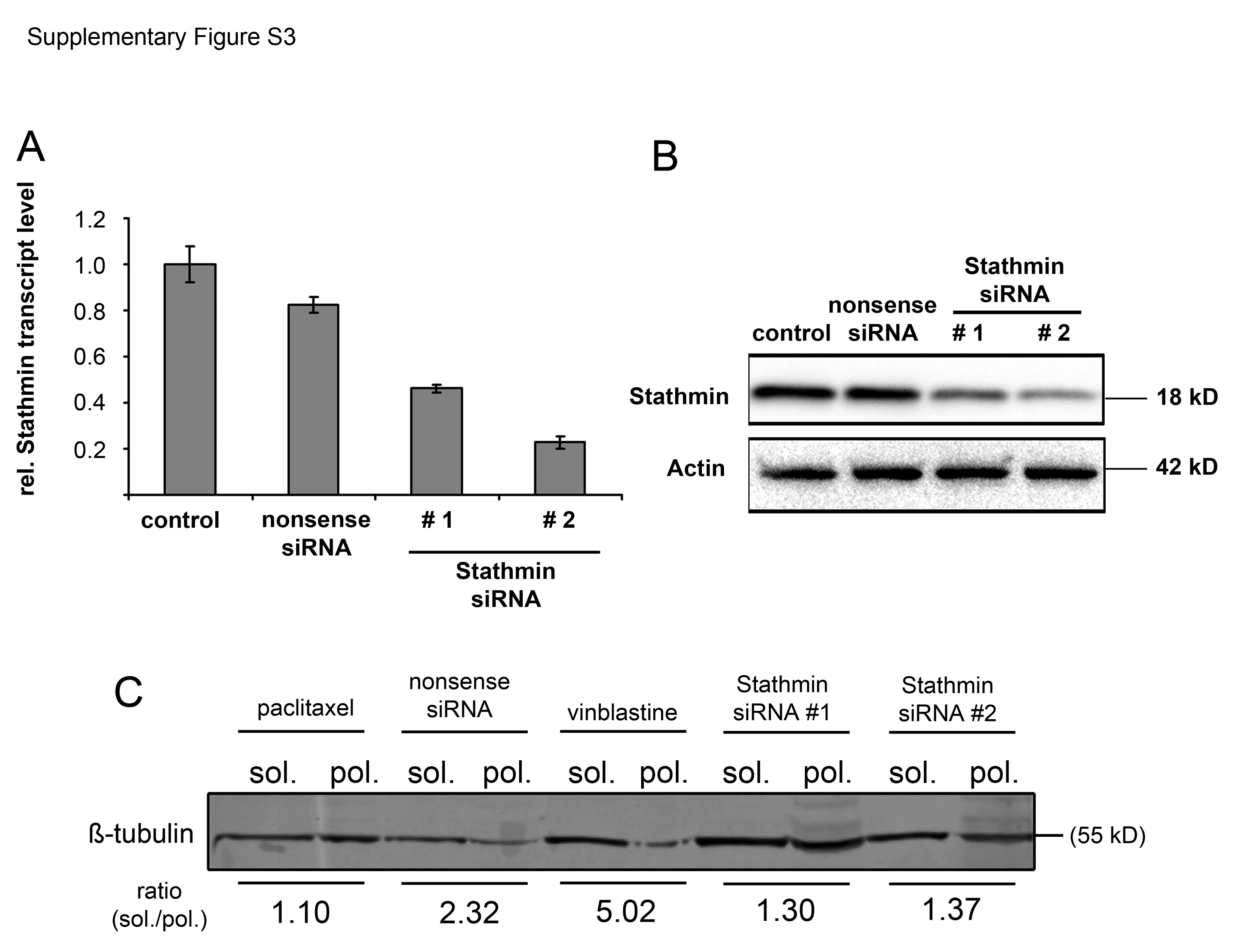

Supplement: Figure S3 — Efficient knockdown of Stathmin transcripts and protein levels. (A) Stathmin transcript levels in primary human keratinocytes were measured by semi-quantitative real-time PCR and (B) western immunoblotting after transient transfection of two Stathmin-specific siRNAs (#1, #2; 20 nM each) for 24 h. Nonsense-transfected cells were used as controls. For normalization, 18S-rRNA and actin were used, respectively. Values represent means +/-SEM; (n=3). (C) The tubulin assay revealed an accumulation of polymerized ß-tubulin for both Stathmin-specific siRNAs as compared with nonsense siRNA-transfected cells. Treatment with microtubule stabilizing paclitaxel (1µM) served as control for tubulin polymerization, while vinblastine (1 µM) served as control for depolymerization. Ratios between soluble (sol.) and polymerized (pol.) tubulin are indicated (high values: low degree of polymerization; low values: high degree of polymerization). (TIF) [file pone.0075075.s003.tif]
