# Supplementary material for: Stathmin Regulates Keratinocyte Proliferation and Migration during Cutaneous Regeneration
Source: PLoS One. 2013 Sep 16;8(9):e75075. doi: 10.1371/journal.pone.0075075 (PMC3774809; doi:10.1371/journal.pone.0075075)
Supplement: Table S1 — Sequences of primers and siRNAs used in this study. (DOCX) [file pone.0075075.s004.docx]

**Supplementary Tables S1**

Supplementary Table S1: Sequences of primers and siRNAs used in this study.

| Name | Gene | Direction | Sequence (5‘ to 3‘ end) |
| --- | --- | --- | --- |
| siRNA #1 | Stathmin | sense | AGG CAA UAG AAG AGA ACA A-dTdT |
| siRNA #2 | Stathmin | sense | AAG AGA AAC UGA CCC ACA A-dTdT |
| siRNA #1 | c-Fos | sense | GGA AAG UGA AUU TGA AUG A-dTdT |
| siRNA #2 | c-Fos | sense | AGG AGA AUC CGA AGG GAA A-dTdT |
| siRNA #1 | c-Met | sense | GCA CAA AGC AAG CCA GAU U-dTdT |
| siRNA #2 | c-Met | sense | GAA CAG AAU CAC UGA CAU A-dTdT |
|  |  |  |  |
| TaqMan Primer | Stathmin | forward | TGC AGA ATA CAC TGC CTG TC |
|  |  | reverse | AGG CAC GCT TCT CCA GTT CT |
| TaqMan Primer | c-Fos | forward | AAC TTC ATT CCC ACG GTC ACT |
|  |  | reverse | TGC AGG TCC GGA CTG GTC GAG |
| TaqMan Primer | c-Met | forward | TTG CTT TGC CAG TGG TGG GAG CA |
|  |  | reverse | GGG GGA GTT GCA GAT TCA GCT GTT |
| TaqMan Primer | 18S | forward | AAA CGG CTA CCA CAT CCA AG |
|  |  | reverse | CCT CCA ATG GAT CCT CGT TA |
