# Supplementary material for: Stathmin Regulates Keratinocyte Proliferation and Migration during Cutaneous Regeneration
Source: PLoS One. 2013 Sep 16;8(9):e75075. doi: 10.1371/journal.pone.0075075 (PMC3774809; doi:10.1371/journal.pone.0075075)
Supplement: Table S2 — List of antibodies used in this study. (DOCX) [file pone.0075075.s005.docx]

**Supplementary Tables S2**

Supplementary Table S2: List of antibodies used in this study.

| Antibody | Isotype | Dilution | Company |
| --- | --- | --- | --- |
| rabbit monoclonal to Stathmin 1 | IgG | 1:750 (WB) 1:100 (IF) 1:100 (IHC) | Abcam |
| rabbit polyclonal to phospho-Stathmin (Ser38) | ------------- | 1:500 (WB)  1:100 (IHC) | Cell Signaling |
| rabbit monoclonal to Met | IgG | 1:750 (WB) | Cell Signaling |
| rabbit monoclonal to phospho-Met (Tyr1234/1235) | IgG | 1:500 (WB) | Cell Signaling |
| rabbit polyclonal  to c-Fos | ------------- | 1:500 (WB) | Abcam |
| mouse monoclonal to Actin | IgG | 1:5000 (WB) | MP Biomedicals |
| rabbit monoclonal to pAkt (Ser473) | IgG | 1:1000 (WB) | Cell Signaling |
| rabbit polyclonal to pErk (Thr202/Thr204) | ------------- | 1:1000 (WB) | Cell Signaling |
| rabbit polyclonal to Erk | ------------- | 1:1000 (WB) | Cell Signaling |
| rabbit polyclonal to Akt | ------------- | 1:1000 (WB) | Cell Signaling |
| mouse monoclonal  to Ki67 | IgG | 1:100 (IHC) | DAKO |
| rat monoclonal  to Ki67 | IgG | 1:100 (IHC) | DAKO |
